# Cabazitaxel operates anti-metastatic and cytotoxic via apoptosis induction and stalls brain tumor angiogenesis

**DOI:** 10.18632/oncotarget.9439

**Published:** 2016-05-18

**Authors:** Ali Ghoochani, Gökce Hatipoglu Majernik, Tina Sehm, Sven Wach, Michael Buchfelder, Helge Taubert, Ilker Y. Eyupoglu, Nicolai Savaskan

**Affiliations:** ^1^ Translational Cell Biology & Neurooncology Laboratory, Department of Neurosurgery, Universitätsklinikum Erlangen, Medical School of The Friedrich-Alexander University (FAU) of Erlangen - Nürnberg, Erlangen, Germany; ^2^ Present Address: Department of Neurosurgery, Medizinische Hochschule Hannover (MHH), Hannover, Germany; ^3^ Department of Urology, Universitätsklinikum Erlangen, Medical School of The Friedrich-Alexander University (FAU) of Erlangen - Nürnberg, Erlangen, Germany; ^4^ BiMECON Ent., Berlin, Germany

**Keywords:** gliomas, neurotoxicity, taxane, angiogenesis, cell death

## Abstract

Taxanes target microtubules and are clinically established chemotherapeutic agents with proven efficacy in human cancers. Cabazitaxel (XRP-6258, Jevtana®) is a second generation semisynthetic taxane with high chemotherapeutic potential in prostate cancer. There, cabazitaxel can overcome docetaxel-resistant prostate cancer. Here, we tested the effects of cabazitaxel on glioma cells, and non-transformed cells such as neurons and astrocytes. Cabazitaxel operates highly toxic in various human glioma cells at nanomolar concentrations. In contrast, primary astrocytes and neurons are not affected by this agent. Cabazitaxel disrupts cytoskeletal F-actin fibers and induces apoptotic cell death in gliomas. Moreover, cabazitaxel displayed highest efficacy in inhibiting glioma cell migration and invasion. Here we demonstrate that cabazitaxel inhibited tumor migration already at 1 nM. We also tested cabazitaxel in the *ex vivo* VOGiM assay. Cabazitaxel stalled glioma growth and at the same time inhibited tumor-induced angiogenesis. In summary, we found that cabazitaxel operates as an apoptosis-inducing gliomatoxic agent with strongest effects on migration and invasive growth. Thus, our report uncovered cabazitaxel actions on gliomas and on the brain tumor microenvironment. These data reveal novel aspects for adjuvant approaches when applied to brain tumor patients.

## INTRODUCTION

Malignant primary brain tumors are the most common type of solid tumors in the CNS affecting children and adults. So far, the prognosis for patients diagnosed with central nervous system tumors such as glioblastomas (GBM) stagnated in recent years, largely due to missing novel efficient and at the same time safe chemotherapeutic agents [1]. The poor prognosis is often ascribed to incomplete tumor resection and the resistance and non-responsiveness of gliomas to current therapeutics. In addition, treatment of these tumors often results in significant long-term disabilities and reduction in quality of life [2, 3]. Therefore, finding new therapies with higher efficacy and at the same time less neurotoxic side effects is essential for improving event-free and overall survival in GBM patients.

During recent years the applicability of drugs or small molecule inhibitors on glioma cells particularly on vascularization and the tumor-immune interaction has been becoming center of interest [4–6]. However, one obstacle in delivering therapeutics to tumors of the central nervous system is tumor-induced angiogenesis which challenges the blood-brain barrier and absorption rate (BBB) [4, 7–9]. The capillary endothelial cells of brain tumors with tight junctions' trait are different and distinguishable from endothelial cells of other tissues in that they lack penetration. This feature has clinical implications in that therapeutic agents are impeded to passively diffuse into the brain [10, 11]. Therefore, discovering small molecule drugs passing the blood-brain barrier more efficient would challenge efficacy in targeting tumor growth rate and pathological tumor angiogenesis.

Cabazitaxel is a second-generation semisynthetic taxane which is currently Food and Drug Agency (FDA) and European Medicines Agency (EMA) approved for treatment of castration-resistant prostate cancer [12]. Indeed, the applicability of first generation taxane therapies (paclitaxel and docetaxel) displayed some limitations. Paclitaxel and docetaxel are high-affinity substrates for ATP-dependent multidrug-resistant pumps [13, 14]. Tumor-induced activation of multidrug-resistant pumps leads to lower penetration and effective concentrations of therapeutic agents across the BBB due to challenged endothelial influx/efflux ratios [15]. In contrast to paclitaxel and docetaxel, the affinity of multidrug-resistant pumps for cabazitaxel is much lower, indicating a better efficacy of cabazitaxel in brain tumors. Moreover, *in vivo* evidence for the distribution of cabazitaxel throughout the brain and the capacity of the substance to get absorbed by endothelial cells of the BBB has recently been shown [16].

Therefore, in this study we tested whether cabazitaxel treatment can successfully fight primary brain tumor growth and whether cabazitaxel can efficiently reverse tumor angiogenesis. In this study we used the *ex vivo* vascular glioma impact method (VOGiM) to investigate the influence of gliomas and chemotherapeutics on the tumor microenvironment and angiogenesis [17]. Our results suggest that application of cabazitaxel does not only prevent glioma growth but also induce enhanced tumor cell death compared to non-tumoral area. Moreover, we show that cabazitaxel treatment reduces tumor-induced angiogenesis while normal non-transformed brain cells and endothelial cells are not affected by this agent.

## RESULTS

### Cabazitaxel reduces glioma cell growth and survival

**Figure 1:**
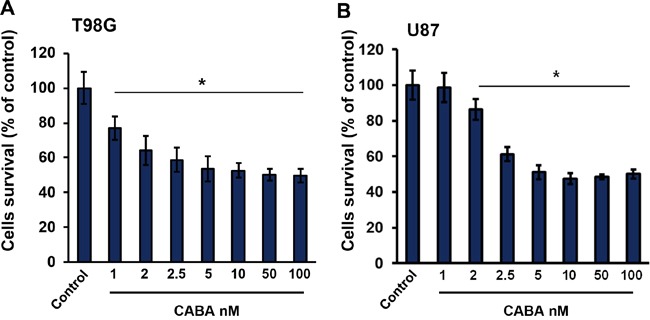
Cell proliferation and survival under cabazitaxel at different concentrations **A.** T98G and **B.** U87 cell lines were treated with 1, 2, 2.5, 5, 10, 50 and 100 nM cabazitaxel for 3 days. MTT assay was implicated to measure cell survival as described in material and methods. Experiment was performed in three independent repetitions. Statistical analysis was performed with One-way ANOVA (*P < 0.05, mean is given ± s.e.m.).

To study the effects of cabazitaxel on brain cancer cell proliferation and survival, we used two human glioma cell lines (T98G and U87) which were treated with a wide range of cabazitaxel concentrations. Glioma cells were seeded in number of 3 × 10^3^ cells in 96-wells plates for a day prior drug application. Next day we treated cells with cabazitaxel for three days at concentrations of 1 to 100 nM in order to investigate its glioma toxicity potential (Figure 1). In T98G and U87 glioma cell lines, cabazitaxel treatment significantly reduced cell survival and proliferation. We found that a concentration of 2.5 nM cabazitaxel was sufficient to inhibit cell proliferation (Figure 1A, 1B). However, 1 nM cabazitaxel was also effective to induce 20% cell death effect on T98G cells (Figure 1A). Taken together, these results demonstrate that cabazitaxel is effective in reducing glioma proliferation although the impact stagnates at 60% even at higher concentrations.

### Cabazitaxel is not toxic to primary neurons and astrocytes

**Figure 2:**
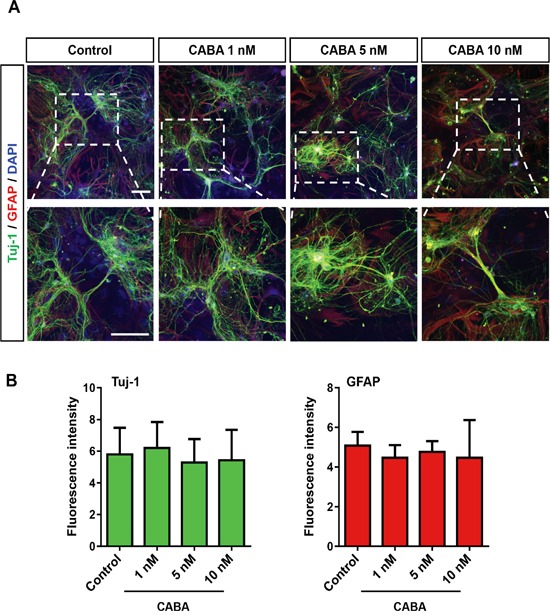
Cabazitaxel is not toxic to primary neurons and astrocytes **A.** Isolated primary hippocampal neurons and astrocytes were treated with 1, 5 and 10 nM cabazitaxel for 3 days. Neurons and astrocyte were stained with anti Tuj-1 (green) and GFAP (red) respectively. Scale bar represents 100 μm. **B.** Quantification of Tuj-1 (β-III tubulin) and GFAP immunostaining (*n = 3*). Statistical analysis was performed with One-way ANOVA (mean is given ± s.e.m.).

In a next step, we isolated rat hippocampal neurons and astrocytes and evaluated whether cabazitaxel impacts selectively on gliomas or is a general toxic agent even for non-transformed brain cells. Therefore, we treated isolated hippocampal neurons and astrocytes with a range of 1 to 10 nM cabazitaxel which appeared to be effective on glioma cells (Figure 1). Cabazitaxel treatment did not adversely change neuronal or astrocyte branches at various concentrations compared to untreated controls (Figure 2A). Both neurons and astrocytes displayed a preserved quality in morphology, branches and expression of Tuj-1 and GFAP neuronal and astrocyte markers, respectively, (Figure 2A) during five days of treatment. All tested concentrations did not significantly challenged both neuronal and astroglial marker expression (Figure 2B). Therefore, these results confirm cabazitaxel as a selective toxic agent for glioma cells which is not toxic for resident brain cells.

### Cabazitaxel induces apoptotic cell death in gliomas

**Figure 3:**
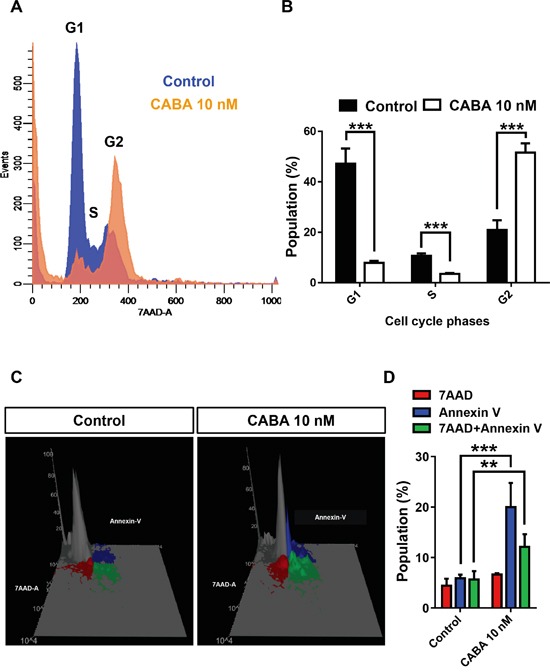
Cabazitaxel induces apoptotic cell death in gliomas **A.** Cell cycle profiling of glioma cells following cabazitaxel (CABA) treatment. Control cell population is given in blue, cabazitaxel treated cell population is given in orange. Note the left shift of CABA treated cells towards the sub-G_1_ phase and the right shift to the G_2_ phase. **B.** Quantification of the cell cycle analysis in human glioma cells. Differences were considered statistically significant with values given as mean ± s.e.m.(*n = 3* independent experiments per group; unpaired t-test, ***P < 0.001). **C.** Fluorescence activated cell sorting-based analysis for apoptosis following cabazitaxel application. Apoptosis was monitored via Annexin V staining given in blue (early apoptosis and membrane integrity) and Annexin V/7AAD double staining given in green (late apoptosis, cell death end stage). The 7AAD pool is shown in red. **D.** Quantification of various apoptotic and cell death fractions. Differences were considered statistically significant with values given as mean ± s.e.m.(*n = 3* independent experiments per group; unpaired t-test, **P < 0.005 ***P < 0.001).

Hence, we investigated the mechanisms of cabazitaxel-induced cell death in gliomas. For this we facilitated human glioma cells and applied effective nanomolar levels of cabazitaxel (Figure 3). Cell cycle profiling of glioma cells following cabazitaxel treatment revealed a left shift of cabazitaxel treated cells towards the sub-G_1_ phase indicating increased apoptotic or necrotic cell death (Figure 3A). Also, cabazitaxel induced a significant right shift to the G_2_ phase which is equivalent to a growth arrest response (Figure 3A, 3B). We further performed fluorescence activated cell sorting-based analysis to uncover the mode of cabazitaxel-induced cell death. Apoptosis was monitored via Annexin V staining and 7AAD staining was used as a marker for the cell death end stage (Figure 3C, 3D). We quantified apoptotic and necrotic cell death and found that cabazitaxel induces mainly apoptotic cell death (Figure 3C, 3D).

### Cabazitaxel suppresses glioma cell migration

**Figure 4:**
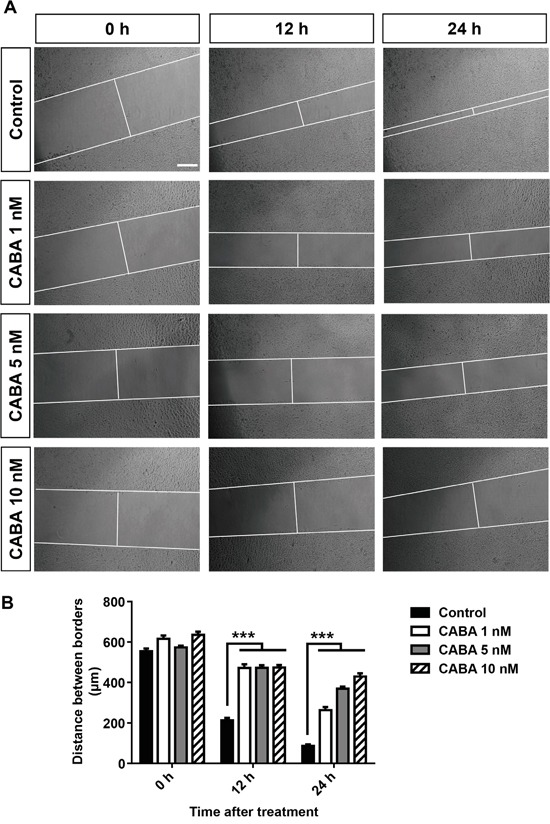
Cabazitaxel stalls glioma cell migration **A.** The distance of scratch borders was measured 0, 12 and 24 hours after treatment with various cabazitaxel concentrations (1, 5 and 10 nM) in human glioma cells. **B.** Quantification of glioma cell migration (*n = 3 per group*). Statistical analysis was performed with One-way ANOVA (values are given as mean ± s.e.m.).

As we could demonstrate that cabazitaxel adversely affects glioma cell survival and proliferation, the question raised whether cabazitaxel is also able to reduce glioma cell migration. Therefore, we performed an assessment for *in vitro* glioma cell migration under cabazitaxel treatment. For this, glioma cells were cultured and allowed to reach more than 90% confluency. Afterwards, mono-layered glioma cells were scratched to create a distinct and quantifiable cell-free area. Along with the scratch, glioma cells were treated with different concentration 1, 5 and 10 nM of cabazitaxel to measure the two-dimensional movement of untreated control samples and cabazitaxel treated glioma cells at different time point 0, 12 and 24 hours after scratch (Figure 4). As representative images illustrate, increasing concentration of cabazitaxel significantly inhibits migration and motility of glioma cells in comparison to untreated controls (Figure 4). The distance of scratch borders measured 86 μm after 24 hours in untreated control samples while this distance was 264, 370 and 430 μm wide after 1 5 and 10 nM cabazitaxel treatment, respectively (Figure 4A). These results demonstrate that cabazitaxel is highly potent to suppress glioma motility and glioma invasion.

### Cabazitaxel inhibits invasive cell growth and disrupts cytoskeletal organization

**Figure 5:**
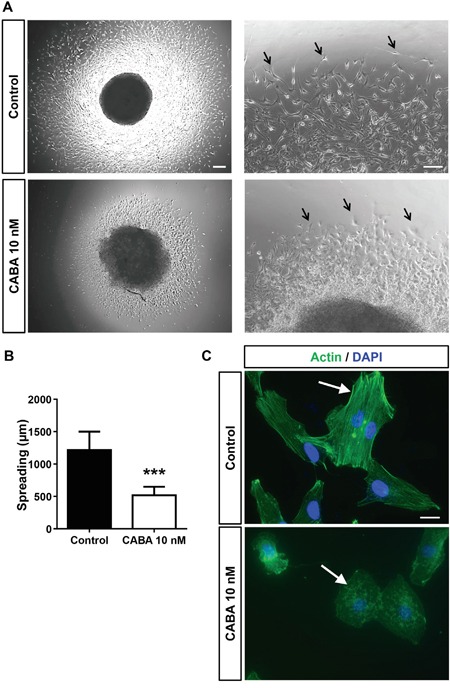
Cabazitaxel inhibits invasive growth and spreading of glioma cells and disrupts F-actin bundles **A.** Glioma cell migration was monitored via the spheroid spreading assay. Left, representative images of human glioma cell clusters from controls and cabazitaxel (CABA) treated gliomas. Asterisks indicate glioma cell migration fronts. **B.** Quantification of the spheroid spreading assay. Three independent experiments were carried out and differences were considered statistically significant with values given as mean ± SD (*n = 5*; One-way ANOVA). **C.** Analysis of cytoskeletal F-actin distribution in human glioma cells under control conditions and following cabazitaxel treatment (CABA). Note the F-actin fiber bundles in controls, whereas following cabazitaxel treatment sole G-actin forms is detectable. Actin staining is given in green, nuclei are displayed in blue.

Next, we investigated the impact of cabazitaxel on glioma invasion. For this we embedded glioma cells in a 3D matrix and observed tumor cell spreading and invasion (Figure 5A, 5B). Following cabazitaxel application glioma cell spreading was significantly reduced (Figure 5B). Moreover, glioma cell morphology appeared challenged with reduced cell membrane extensions compared to untreated cells (Figure 5A). We further investigated this phenomenon and monitored the cytoskeleton following cabazitaxel application (Figure 5C). Under control conditions glioma cells showed regularly organized F-actin bundles in the cytoskeleton (Figure 5C). In contrast, glioma cells following cabazitaxel incubation showed disrupted actin fibers with G-actin like formations in the cytoskeleton (Figure 5C). Thus, our data show that cabazitaxel acts on the actin cytoskeleton and inhibits tumor invasion.

### Cabazitaxel stalls tumor growth in the *ex vivo* VOGiM assay

**Figure 6:**
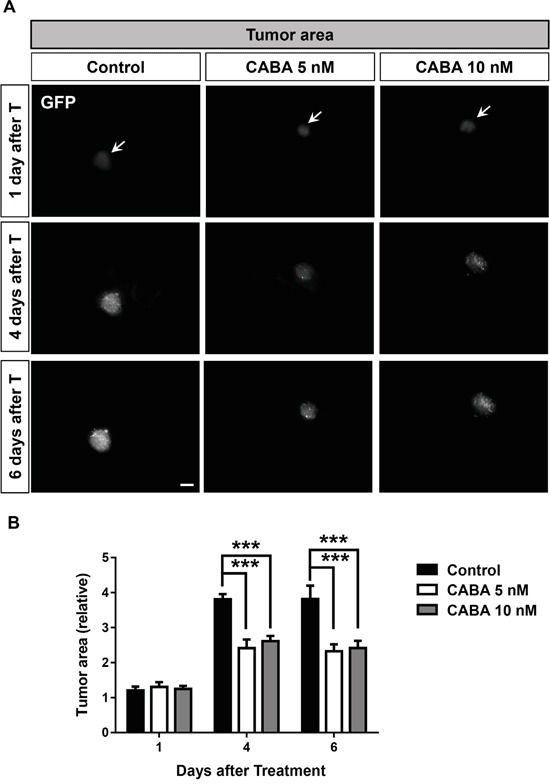
Cabazitaxel reduces glioma growth and tumor size in *ex vivo* VOGiM cultures **A.** Tumor growth is illustrated in an *ex vivo* Organotypic Glioma Impact Model (VOGiM) as described in material and methods for 6 days after treatment. The rat brain slices were treated with 5 and 10 nM cabazitaxel concentrations. Control samples were treated with DMSO as vehicle. **B.** Tumor size measurement with Image J software at 1, 4 and 6 days after treatment (*n = 9*). Scale bar represents 500 μm. Statistical analysis was performed with One-way ANOVA (*P < 0.05, **P < 0.01, *** P < 0.001 and error bars represent mean ± s.e.m.).

Hence, we investigated the impact of cabazitaxel in a complex tumor microenvironment. For this we acquired the *ex vivo* vascular glioma impact method (VOGiM) to measure tumor indexes and characteristics such as volume, death and peritumoral angiogenesis (see Material and methods). The VOGiM assay allows to study whether cabazitaxel negatively influences tumor growth in *ex vivo* culture. Therefore, rat brains from postnatal day four were prepared and dissected in sagittal plane. After 24 hours incubation step of brain slices, GFP positive glioma cells were implanted into the cortex. After overnight incubation, tumor implanted brain slices were treated with 5 nM and 10 nM cabazitaxel. One day after treatment, both 5 nM and 10 nM cabazitaxel (CABA) concentrations did not change tumor size compared to untreated control samples (Figure 6A). However, longer treatment durations of both cabazitaxel concentrations for four to six days significantly led to tumor growth reduction in comparison to untreated control samples (Figure 6A, 6B). After four days treatment, quantification revealed increased tumor reduction (aprox. 50%) in cabazitaxel treated brains compared to untreated controls (Figure 6B). These data confirm well the potency of cabazitaxel on suppressing glioma growth rate in *ex vivo* brain sections (Figure 6B). Noteworthy is the fact, that both 5 nM and 10 nM cabazitaxel were effective in inhibiting tumor growth, indicating concentrations which are reachable also in patients. Taken together, the results confirm the suppressive cabazitaxel influence on tumor growth in both *in vitro* and *ex vivo* cultures.

### Cabazitaxel induces tumor cell death in *ex vivo* VOGiM cultures

**Figure 7:**
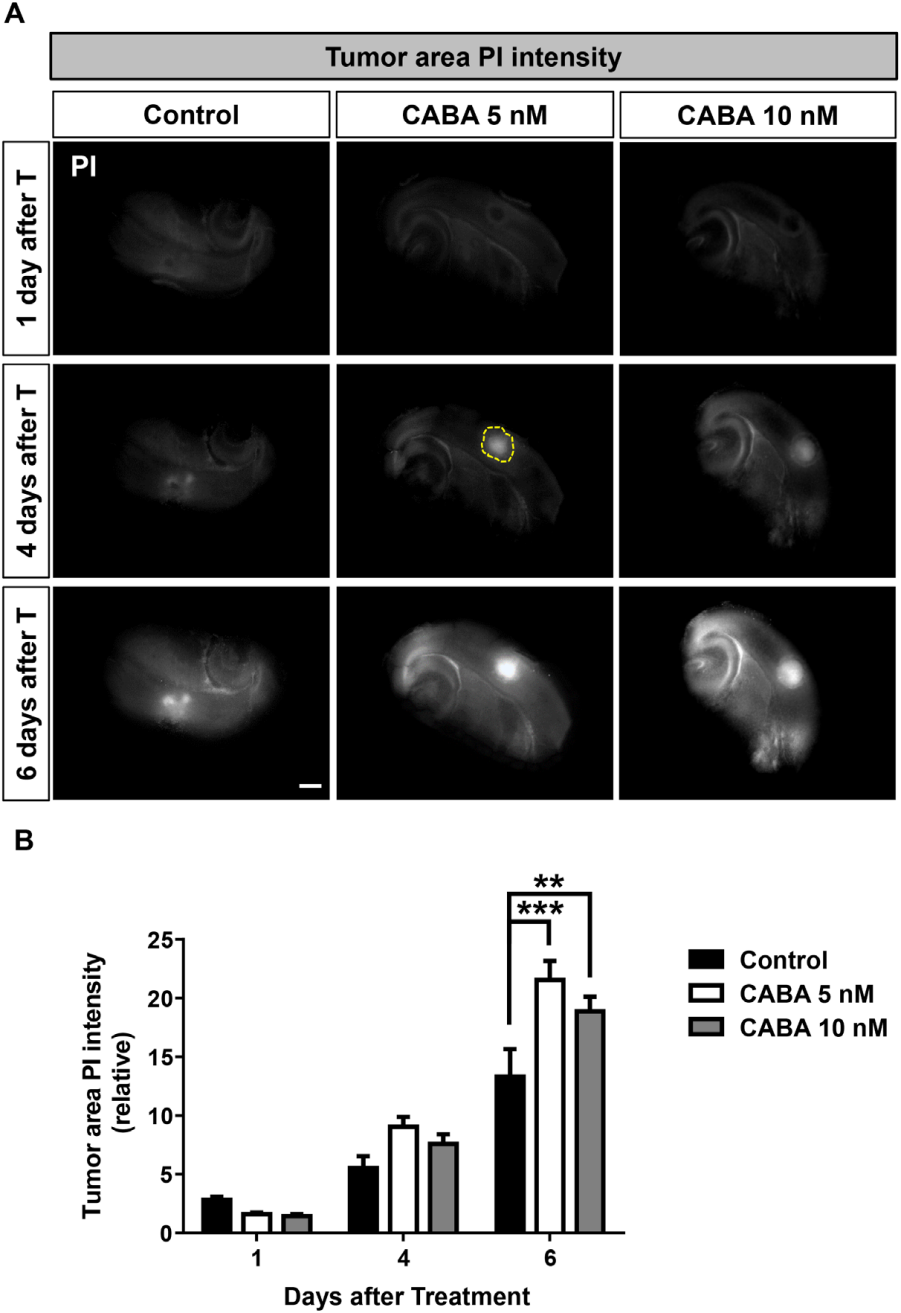
Cabazitaxel induces specifically tumor cell death in the brain microenviroment **A.** Tumor cell death analysis which illustrated by PI intensity in the tumor area at 1, 4 and 6 days after treatment. The rat brain slices were treated with 5 nM or 10 nM cabazitaxel. Control samples were treated with DMSO as vehicle. Yellow circle displays tumor area. **B.** Quantification of PI integrated intensity with Image J software at 1, 4 and 6 days after treatment (*n = 9*). Scale bar represents 500 μm. Statistical analysis was performed with One-way ANOVA (*P < 0.05, **P < 0.01, *** P < 0.001 and error bars represent mean ± s.e.m.).

We could show in the VOGiM assay that cabazitaxel reduced implanted tumor size in a complex tumor microenvironment. The question remained whether these tumor reducing effects of cabazitaxel were due to growth arrest or apoptosis and secondary necrosis. Therefore, we simultaneously monitored tumor cell death in the VOGiM. Cell death measurements via the marker propidium iodide (PI) demonstrated increasing cell death in the tumor area corresponding to higher cabazitaxel concentrations (Figure 7A, yellow circle). After four days of treatment with 5 nM and 10 nM cabazitaxel were already effective in increasing tumor cell death compared to untreated control samples (Figure 7A, 7B). However, cabazitaxel treatment with both levels resulted in up to 25% tumor cell death increase after six days (Figure 7A, 7B). Moreover, both concentrations illustrated similar cell death impact which confirms data on tumor size reduction following cabazitaxel application (Figure 7B). Interestingly, cabazitaxel treatment did not significantly effect on cell death of non-tumoral area (Figure 7A). Taken together we could show that cabazitaxel enables selectively cell death in the tumor area.

### Cabazitaxel reduces peritumoral vascularization

**Figure 8:**
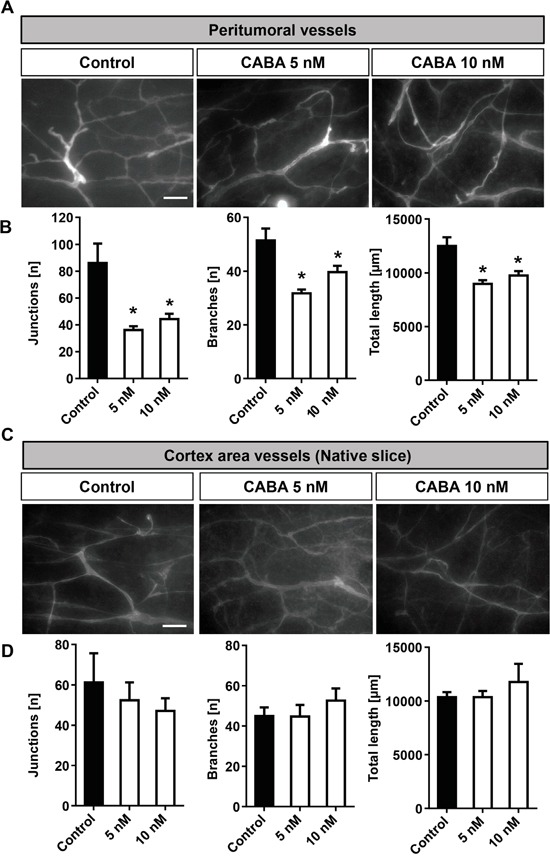
Cabazitaxel reduces tumor angiogenesis *ex vivo* **A.** Laminin staining for the detection of vascularization in peritumoral area under cabazitaxel treatment. Rodent brain slices were implanted with GFP positive F98 cells and were treated with 5 and 10 nM cabazitaxel for 6 days. **B.** Quantification of vascularization (number of junctions, number of branches and total vessels length in peritumoral area. **C.** Cabazitaxel does not affect normal vessel architecture. Laminin staining for vascularization in cortex of native brain slices under 5 and 10 nM cabazitaxel treatment. **D.** Quantification of physiological vascularization following cabazitaxel treatment compared to controls (number of junctions, number of branches and total vessels length in the cortex of native brain slices. Scale bar represents 50 μm (*P < 0.05, **P < 0.01, *** P < 0.001 and error bars represent mean ± s.e.m.).

The ability of a tumor to stimulate new blood vessel formation is one hallmark of malignant gliomas. The oxygen and nutrients supplied by the vasculature are crucial for tumor expansion and invasion [18, 19]. Therefore, we sought to identify whether cabazitaxel is also able to affect peritumoral vessels formation in brain tumors in the *ex vivo* VOGiM assay. Therefore, we implanted glioma cells into brain slices after which we treated tumor-implanted brain sections with 5 and 10 nM cabazitaxel. After running the VOGiM for 6 days, slices were fixed and vessels stained for laminin (Figure 8A). To assess vascularization, we did analyze in particular three vascular indexes: number of junctions, number of branches and total length (see also Material and methods).

Glioma implantation induced peritumoral angiogenesis and tumoral vascularization (Figure 8A–8D). Following treatment, visualization of vessels in peritumoral area displayed clearly a reduction in vascularization in cabazitaxel treated samples (Figure 8A, 8B). The quantification of vessels also demonstrated all parameters: number of junctions, number of branches and total length were decreased under both 5 and 10 nM cabazitaxel concentrations compared to untreated control samples (Figure 8B).

Since our data revealed that cabazitaxel selectively affects the tumor microenvironment, we proceeded with analyzing the impact of cabazitaxel on normal brain vascularization. Therefore, native rat brain sections were treated with cabazitaxel for 6 days. To analyze vascularization we quantified the same vessel parameters as measured in the peritumoral area of the VOGiM setting. The results revealed that cabazitaxel did not significantly change physiological vascularization parameters (Figure 8C, 8D). In conclusion, our results indicate that cabazitaxel is selectively impacting on peritumoral and tumor vessels whereas normal vascularization is not affected.

## DISCUSSION

Malignant gliomas are intrinsic brain tumors and are the most common and lethal primary brain tumors derived from glial cells [20]. Moreover, gliomas have a high tendency to spread out into neighboring regions where they form satellite colonies [21]. In cases where neurosurgery is not applicable, chemotherapy is the first line primary treatment method for gliomas [5, 22]. However, the characteristics of tumor vascularization represent an obstacle for delivering therapeutics to tumors of the central nervous system due to challenged permeability and blood-brain barrier function [2, 23]. The capillary endothelial cells of brain tumors are pathologically challenged in the way they lead to lack of penetration and preventing the passive diffusion of therapeutics into the brain [10, 11]. Therefore, discovering and application of agents or small molecules with high ability to passively diffuse across the blood-brain barrier would potentiate glioma therapy. Cabazitaxel is a second-generation taxane which has been recently used for prostate cancer treatment [12, 16, 24]. In fact, the affinity of multidrug-resistance pumps for first generation taxanes is an obstacle narrowing the efficacy in brain tumors. However, cabazitaxel's affinity to these pumps has been shown to be rather low [16, 25]. Moreover, cabazitaxel distributes with even pattern throughout the brain [16]. Therefore, these features make cabazitaxel a potential option for glioma first and second line treatment.

In this study, we showed that cabazitaxel is a potent cytotoxic agent for gliomas. Our results revealed that cabazitaxel treatment significantly reduced glioma survival and proliferation. Moreover, we demonstrated that low concentration of cabazitaxel was sufficiently enough to influence glioma cells. In addition, treatment of primary neurons and astrocytes with cabazitaxel did not affect cell survival or morphology. Moreover, our results also illustrated that cell survival did not change in non-tumoral areas under cabazitaxel treatment in the VOGiM assay. However, concerning unintended side actions there have been published two opposing data sets. In the study of Girard and colleagues [2] the maximum tolerated dose was determined for C57BL6 mice. Mice with Smo/Smo flank allograft tumors were treated intraperitoneal with 9, 15, or 25 mg/kg cabazitaxel for 3 days in total. Although at 25 mg/kg cabazitaxel application mice lost significantly body weight, all mice recovered from the weight loss and returned to their starting weight after 20 days [2]. In contrast, Karavelioglu and colleagues [26] reported on intraperitoneal application of cabazitaxel in Wistar rats. These authors found that cabazitaxel applied at 1.0 mg/kg body weight or higher (i.p. injection per week for consecutive 4 weeks) induced neurotoxic effects in rat brains with increased apoptosis. However, in clinical phase I trial including 21 patients with cervical, colorectal, endometrial and lung cancer received escalating dosages of cabazitaxel [31]. Eventually, a safe drug level of 25 mg/m^2^ of cabazitaxel was recommended for use in future clinical trials.

Our data clearly show that in a setting with simultaneous presence of tumor and non-transformed cells, cabazitaxel has a higher impact on tumor cells without signs of neurotoxicity. Future studies are required to investigate in more detail the best route of cabazitaxel application and species differences in order to clarify opposing reports.

Cancer cells become accustomed to angiogenesis and for this require migratory potential as a way of guaranteeing an adequate nutrients supply [27]. In particular migration, velocity and invasive expansion are essential abilities of cancer cells allowing them to change position within compartments and tissues [28]. Our results reveal that cabazitaxel inhibits glioma cell migration and invasion. We could further show that cabazitaxel reduces tumor growth in the brain microenvironment. Tumor-implanted brain slices were treated with cabazitaxel and showed about 50% reduction compared to control samples. Recent studies indicate that tumor microtubules are required for glioma invasion [29]. Cabazitaxel is in fact a microtubule inhibitor [30] which may explain the anti-migratory function of cabazitaxel. In addition, we found strong tumor-angiogenic inhibitory effects of cabazitaxel. This is notable since normal vascularization was not affected by Cabazitaxel. This is an important feature since the treatment of GBM is a delicate balance between aggressive elimination of cancer cells and tumor vessels and preserving brain function and protection of healthy neural tissue [1].

In conclusion, this report represents cabazitaxel as an efficient and selective compound to stall tumor growth and tumor angiogenesis with low cytotoxicity for brain cells. These drug features are promising for applying cabazitaxel in clinical settings.

## MATERIALS AND METHODS

### Cell culture

T98G human glioma cell line and U87 human glioma cell line were obtained from ATCC/LGC-2397 (Germany) and both cell lines were cultured under standard humidified conditions (37°C, 5% CO2) with Dulbecco's Modified Eagle Medium (DMEM; Biochrom, Berlin, Germany) supplemented with 10% fetal bovine serum (Biochrom, Berlin, Germany), 1% Penicillin/Streptomycin (Biochrom, Berlin, Germany) and 1% Glutamax (Gibco/Invitrogen, California, USA). 80% confluence glioma cells were washed with PBS for 3 min and then treated with trypsin 0.05% at 37°C for 3 min for dissection and then collected by gentle pipetting.

### Survival assay

Cell survival was assessed by MTT assay. For this, 3 × 10^3^ cells (T98G or U87 cell lines) were seeded for 3 days and incubated along with cabazitaxel (MedKoo Biosciences, USA) treatment. Cabazitaxel was added at 1, 2.5, 5, 10, 50, and 100 nM final concentrations. To measure survival, 3 hours before the desired time point, 10 μl of MTT solution (5 mg/ml in PBS) was added into each well and cells were incubated at 37°C. After 3 hours incubation, the medium was removed and 100 μl of isopropanol / 37% HCL (Roche, Germany) was added into each well. The absorbance was detected at 570 nm with a Microplate Reader (TECAN).

### Apoptosis and cell cycle analysis

Cell cycle analysis was performed according to Sehm et al. [32]. Briefly, cells were seeded on culture dishes and kept under standard conditions for 3 days. Hence, cells were isolated, washed thrice in PBS and resuspended in hypolysis buffer containing 0.1% sodium citrate, 0.1% Triton X-100, and 100 g/ml RNAse A. Nuclei were then stained with 7AAD and analyzed by fluorescence-activated cell sorting (FACS) [32]. Cell cycle phases were analyzed with Cellsoft software.

### Migration assay

To perform glioma migration, glioma cells lines were allowed to grow to 10% FBS containing DMEM medium to confluence (more than 90%) as described previously [33]. One mm wide cell-free scratch wound was made across the cell layer by a sterile pipette tip. After washing twice with serum-free medium, medium was changed to new medium containing 5 and 10 nM cabazitaxel and immediately imaged after scratching as 0 h. Plates were photographed after 12 and 24 hours at the same location of initial image to measure glioma migration. Image J software was used for further analysis.

### Spheroid invasion assay

For the spheroid assay cells were embedded in methyl-cellulose and the invasion front was quantified as described recently [33].

### *Ex vivo* VOGiM brain slice cultures and tumor implantation

An extended description of the VOGiM assay has been presented previously [17]. Briefly, postnatal Wistar rat pups (Charles River, Boston, MA, USA) were decapitated. Brains were cautiously prepared and fixed to cut horizontally the brain into 350 μm thick slices. Slices were incubated for one day in culture medium. Next day, GPF-expressing F98 cells were implanted into the brain slices. After 24 h, brain slices were treated with 5 and 10 nM cabazitaxel. In parallel, brain slices were also treated with propidium iodide to measure cell death. Brain slices were photographed 1, 4 and 6 days after tumor implantation to monitor tumor volume and death [17].

### Immunofluorescence staining

Primary neurons and astrocytes were fixed with 4% paraformaldehyde (Sigma-Aldrich) for 20 min in room temperature, washed with PBS twice followed by permeabilization for 30 min with 0.4% Triton-X (Sigma-Aldrich) in PBS. Primary β-tubulin III (Tuj-1) (Promega) and GFAP antibodies (Dako) were diluted in 0.4% Triton-X (Sigma-Aldrich) + 3% FCS as the blocking solution and subsequently incubated with the samples for overnight at 4°C. Samples were washed with PBS and subsequently labeled with Alexa Fluor 488 and 568 (Invitrogen/LifeTechnologies) as secondary antibodies for 60 min at 37°C. Hoechst dye (Invitrogen/LifeTechnologies and Sigma Aldrich) was used to stain nuclei.

For vascular brain staining, brain slices were fixed with 4% paraformaldehyde for 60 min in room temperature, washed with PBS twice followed by permeabilization for 30 min with 0.4% Triton-X in PBS. Then, brain slices were incubated with anti-laminin antibody which was prepared with 0.4% Triton-X + 3% FCS for overnight at 4°C. After washing with PBS, samples were incubated with Alexa Fluor 568 for another overnight at 4°C. Hoechst dye (Sigma Aldrich Chemicals) was used to stain nuclei.

## Abbreviations

CABA: Cabazitaxel
EMA: European Medicines Agency
FDA: Food and Drug Administration
GFP: green fluorescent protein
PI: propidium iodide
VOGiM: Vascular organotypic glioma impact and invasion model
